# The Absence of Serotonin in the Brain Alters Acute Stress Responsiveness by Interfering With the Genomic Function of the Glucocorticoid Receptors

**DOI:** 10.3389/fncel.2020.00128

**Published:** 2020-06-01

**Authors:** Giulia Sbrini, Paola Brivio, Polina Mineva Peeva, Mihail Todiras, Michael Bader, Natalia Alenina, Francesca Calabrese

**Affiliations:** ^1^Department of Pharmacological and Biomolecular Sciences, Università Degli Studi di Milano, Milan, Italy; ^2^Cardiovascular and Metabolic Diseases, Max-Delbrück-Center for Molecular Medicine (MDC), Berlin, Germany; ^3^Charite-University Medicine, Berlin, Germany; ^4^Institute of Translational Biomedicine, St. Petersburg State University, St. Petersburg, Russia

**Keywords:** serotonin, tryptophan hydroxylase, HPA axis, glucocorticoid, clock genes, rats, prefrontal cortex

## Abstract

Alterations in serotonergic transmission have been related to a major predisposition to develop psychiatric pathologies, such as depression. We took advantage of tryptophan hydroxylase (TPH) 2 deficient rats, characterized by a complete absence of serotonin in the brain, to evaluate whether a vulnerable genotype may influence the reaction to an acute stressor. In this context, we investigated if the glucocorticoid receptor (GR) genomic pathway activation was altered by the lack of serotonin in the central nervous system. Moreover, we analyzed the transcription pattern of the clock genes that can be affected by acute stressors. Adult wild type (TPH2^+/+^) and TPH2-deficient (TPH2^−/−^) male rats were sacrificed after exposure to one single session of acute restraint stress. Protein and gene expression analyses were conducted in the prefrontal cortex (PFC). The acute stress enhanced the translocation of GRs in the nucleus of TPH2^+/+^ animals. This effect was blunted in TPH2^−/−^ rats, suggesting an impairment of the GR genomic mechanism. This alteration was mirrored in the expression of GR-responsive genes: acute stress led to the up-regulation of GR-target gene expression in TPH2^+/+^, but not in TPH2^−/−^ animals. Finally, clock genes were differently modulated in the two genotypes after the acute restraint stress. Overall our findings suggest that the absence of serotonin within the brain interferes with the ability of the HPA axis to correctly modulate the response to acute stress, by altering the nuclear mechanisms of the GR and modulation of clock genes expression.

## Introduction

Psychiatric pathologies in humans have a multifactorial etiology with genetic and environmental factors that may interact thus inducing the manifestation of a wide range of symptoms (Akiskal and McKinney, 1973, 1975). Among the genetic factors that can predispose to the development of psychiatric illnesses, such as depression, alterations in the serotonergic system are the most studied not only at clinical level but also in preclinical studies (Caspi et al., 2003; Calabrese et al., 2013; Brivio et al., 2018; Ottenhof et al., 2018). Interestingly, serotonergic projections innervate the paraventricular nucleus of the hypothalamus as well as the brain regions involved in the stress-induced activity of the HPA axis, including the prefrontal cortex (PFC) (Pan and Gilbert, 1992; Van de Kar et al., 2001; Herman et al., 2003).

Serotonin synthesis starts from the amino acid tryptophan through the action of the rate-limiting enzyme tryptophan hydroxylase (TPH) (Fitzpatrick, 1999). This enzyme exists in two isoforms: TPH1 expressed at the periphery, and TPH2, expressed in the raphe nuclei in the brain and responsible for the production of serotonin at the central level (Walther et al., 2003). Accordingly, animal models with genetic deletion of TPH2 show reduced levels of serotonin in the brain (Mosienko et al., 2015).

As mentioned above, also environmental factors play a key role in the manifestation of mood disorders. Chronic stress is one of the main precipitating factors, that negatively affect behaviors (Breslau and Davis, 1986; Lupien et al., 2018). On the contrary, acute stressors can have a positive impact on the brain leading to the activation of neuroplastic mechanisms and the transcription of activity-dependent genes (Brivio et al., 2020). In this context, we recently demonstrated that despite a normal corticosterone–release pattern upon acute stress, TPH2-deficiency in rats results in an up-regulation of the neurotrophin BDNF in basal conditions, but a blunted response to acute stress (Brivio et al., 2018).

Here, we took advantage of the TPH2-deficient (TPH2^−/−^) rats (Kaplan et al., 2016), characterized by a complete absence of serotonin in the brain, to evaluate the impact of central serotonin deficiency on the acute stress response. Hence, we evaluated if the molecular changes that physiologically occur after stress exposure could be altered by the absence of serotonin in the brain focusing on the hypothalamic-pituitary-adrenal (HPA) axis, one of the main mechanisms that allow a proper response to stressful events (McEwen, 2007). In particular, we analyzed the genomic pathway activation that occurs after the translocation into the nucleus of the glucocorticoid receptors (GRs) (Trapp et al., 1994) and the subsequent transcription of genes carrying the glucocorticoid responsive element (GRE) in their regulatory DNA regions. Interestingly, genes involved in the control of circadian rhythms, period circadian regulator (*Per1*) and *Per2*, are among the glucocorticoid responsive genes, and their expression is known to be affected by acute stress exposure (Yamamoto et al., 2005) as well as by corticosterone levels in the blood (Nader et al., 2010). Therefore, we also analyzed the gene expression profile of the main players involved in the control of the clock gene machinery in TPH2^−/−^ and wild type (TPH2^+/+^) rats after the acute challenge. All molecular analyses were performed in the PFC, a brain region connected not only to serotonin functions but also deeply affected by stress exposure (Duman and Monteggia, 2006; Stuss and Knight, 2009; Brivio et al., 2020).

## Materials and Methods

### Animals

Animals were kept at the animal facility of Max-Delbrück Center for Molecular Medicine under standardized conditions with an artificial 12/12-h light/dark cycle (lights on at 6 a.m.), room temperature of 22°C, and approximately 80% humidity with access to food (Ssniff, Soest, Germany) and water *ad libitum*. All procedures were approved by the ethical committee of the local government (LAGeSo, Berlin, Germany).

Twelve to 14 weeks old male TPH2-deficient (TPH2^−/−^) (Kaplan et al., 2016) and wild type (TPH2^+/+^) rats on the Dark Agouti background were used in the experiments. Animals heterozygous for the TPH2 mutation (10 bp deletion in exon 7) were bred to generate experimental groups of both genotypes. To genotype offspring, genomic DNA from ear biopsies was amplified with primers TPH2ZFN_FW 5^′^-CCC TTC TCC ACA GAA GTG CT and TPH2ZFN_REV 5^′^-GGC CTT TAG GTC CTG AGG TT and the resulting PCR fragment was digested with the restriction nuclease Mnl1, which cuts exclusively the PCR products of the WT allele.

### Stress Procedure

Adult male TPH2^+/+^ and TPH2^−/−^ rats were exposed to acute restraint stress as previously described (Brivio et al., 2018; Calabrese et al., 2020). Briefly, rats were placed in an air-accessible apparatus for 1 h. The size of the container was similar to the size of the animal, making the rats almost immobile. Stressed animals (*n* = 5 per genotype) were sacrificed immediately at the end of the stress session. Unstressed animals (*n* = 13) served as a control group.

### Brain Tissue Collection

Animals were sacrificed by decapitation and the PFC was rapidly dissected, frozen on dry ice and stored at −80°C for the molecular analyses. Dissections were performed according to the atlas of Paxinos and Watson (Paxinos and Watson, 2007). PFC was dissected from 2-mm-thick slices [PFC defined as Cg1, Cg3 and IL sub-regions corresponding to plates 6–9 (approximate weight 8 mg)]. The left hemisphere was taken for protein whereas the right was taken for RNA.

### RNA Preparation and Gene Expression Analysis by Quantitative Real-Time PCR

Total RNA was isolated by a single step of guanidinium isothiocyanate/phenol extraction using PureZol RNA isolation reagent (Bio-Rad Laboratories, Italy) according to the manufacturer’s instructions and quantified by spectrophotometric analysis.

The samples were then processed for real-time polymerase chain reaction (RT-PCR) to assess the expression of Growth Arrest And DNA Damage Inducible β (*Gadd45β*), Serum/Glucocorticoid Regulated Kinase (*Sgk1*), Nuclear Receptor Subfamily four Group A Member 1 (*Nr4a1*), Dual Specificity Phosphatase 1 (*Dusp1*), S100A10 (*P11*), Forkhead box O1 (*FoxO1*), FKBP Prolyl Isomerase 5 (*Fkbp5*), TSC22 Domain Family Member 3 (*Gilz*), *Per1*, *Per2*, cryptochrome circadian regulator (*Cry1*), *Cry2*, nuclear receptor subfamily 1, group D member 1 (*Rev-erb*α), nuclear receptor subfamily, group D, member 2 (*Rev-erb*β) (primer and probes sequences are listed in Tables s 1A,B). RNAs were treated with DNase (Thermoscientific, Italy) to avoid DNA contamination. Gene expression was analyzed by TaqMan qRT-PCR one-step RT-PCR kit for probes (Bio-Rad laboratories, Italy). Samples were run in 384 well formats in triplicate as a multiplexed reaction with a normalizing internal control (*36b4*).

**Table 1A:** Sequences of forward and reverse primers and probes used in Real-time PCR analyses and purchased from Eurofins MWG-Operon.

Gene	Forward primer	Reverse primer	Probe
*36b4*	TTCCCACTGGCTGAGGT	CGCAGCCGCTGC	AAGGCCTTCCTGGCCGATCCATC
*Sgk1*	GACTACATTAATGGCGGAGAGC	AGGGAGTGCAGATAACCCAAG	TGCTCGCTTCTACGCAGC
*Dusp1*	TGTGCCTGACAGTGCAGAAT	ATCTTTCCGGGAAGCATGGT	ATCCTGTCCTTCCTGTACCT
*P11*	AGAGTGCTCATGGAAAGGGA	AGCTCTGGAAGCCCACTTTT	ATAATGAAAGACCTGGACCAGTGC
*FoxO1*	GAGTGGATGGTGAAGAGTGTG	GGACAGATTGTGGCGAATTG	TCAAGGATAAGGGCGACAGCAACAG
*Fkbp5*	GAACCCAATGCTGAGCTTATG	ATGTACTTGCCTCCCTTGAAG	TGTCCATCTCCCAGGATTCTTTGGC
*Gilz*	CGGTCTATCAACTGCACAATTTC	CTTCACTAGATCCATGGCCTG	AACGGAAACCACATCCCCTCCAA
*Per1*	AGAGCTGAGTCCTTGCCATT	TGGCTGATGACACTGATGCA	AGCGGAGTTCTCACAGTTCA
*Per2*	TTGTGCCTCCCGATGATGAA	AGTGGGCAGCCTTTCGATTA	GTACATCACACTGGACACTAGC
*Cry1*	TCAATCCACGGAAAGCCTGT	CCACAAACAACCCACGCTTT	GGAACCCCATCTGTGTTCAA
*Cry2*	TAGTCCACGCCAATGATGCA	TGCCCAAACTGAAAGGCTTC	TCTATGAGCCCTGGAATGCT
*Rev-erbα*	ACGTCCCCACACACTTTACA	ACAAGTGGCCATGGAAGACA	GGCACCAGCAACATTACCAA
*Rev-erbβ*	ACGGATGAGTGTTTCCTGCA	AGCGACGAGGAAATGAGCTT	TTCTGGTGTCTGCAGATCGA

**Table 1B:** Probes purchased from Life Technologies, which did not disclose the sequences.

Gene	Accession Number	Assay ID
*Gadd45β*	BC085337.1	Rn01452530_gI
*Nr4a1*	BC097313.1	Rn01533237_m1

Thermal cycling was initiated with an incubation at 50°C for 10 min (RNA retrotranscription) and then at 95°C for 5 min (TaqMan polymerase activation). After this initial step, 39 cycles of PCR were performed. Each PCR cycle consisted of heating the samples at 95°C for 10 s to enable the melting process and then for 30 s at 60°C for the annealing and extension reactions. A comparative cycle threshold (Ct) method was used to calculate the relative target gene expression.

### Protein Extraction and Western Blot Analysis

Western blot analysis was used to investigate GR, Myc-associated zinc finger protein 1 (MAZ1) and Specificity Protein 1 (SP1) protein levels in the nuclear fraction, in the cytosolic compartment and the whole homogenate. Tissues were manually homogenized using a glass-glass potter in a pH 7.4 cold buffer containing 0.32 M sucrose, 0.1 mM EGTA, 1 mM HEPES solution in the presence of a complete set of proteases (Roche) and phosphatase (Sigma-Aldrich) inhibitors. The total homogenate was centrifuged at 1,000 *g* for 10 min at 4°C to obtain a pellet enriched in nuclear components, which was suspended in a buffer [20 mM HEPES, 0.1 mM dithiothreitol (DTT), 0.1 mM EGTA] with protease and phosphatase inhibitors. The supernatant was further centrifuged at 10000 *g* for 10 min at 4°C to eliminate the membrane fraction and the resulting supernatant corresponding to the cytosolic fraction was conserved for the protein analyses. The purity of these fractions was previously reported (Brivio et al., 2019). Total protein content was measured according to the Bradford Protein Assay procedure (Bio-Rad Laboratories), using bovine serum albumin (BSA) as a calibration standard. Equal amounts of protein were run under reducing conditions on 10% SDS-polyacrylamide gels and then electrophoretically transferred onto Polyvinylidene Difluoride (PVDF) or nitrocellulose membranes (GE Healthcare Life Sciences). The blots for GR were blocked with BSA in TBS+0.2% sodium azide, while the others (MAZ1, SP1 and β-actin) with 5% nonfat dry milk and then were incubated with the proper primary antibodies [GR: 1:500 (ThermoFisher), 4° overnight (O/N); MAZ1: 1:500 BSA 5% 4°C O/N; SP1: 1:250 BSA 5% 4°C O/N β-actin: 1:10,000 (Sigma), room temperature (RT) 45 min]. Membranes were then incubated with the appropriate secondary antibody (GR: anti-rabbit, 1:2,000, RT, 1 h; MAZ1: anti-rabbit 1:1,000, RT 1 h; SP1: anti-rabbit 1:1,000 RT 1 h; β-actin: anti-mouse, 1:10,000, 45 min). Immunocomplexes were visualized by chemiluminescence using the Western Lightning Clarity ECL (Bio-Rad Laboratories) and the Chemidoc MP imaging system (Bio-Rad Laboratories). GR levels were quantified by the evaluation of band densities, normalized to the β-actin (ImageLab, Bio-Rad Laboratories).

### Statistical Analysis

All the analyses were conducted by using “IBM SPSS Statistics, version 24.” Results were analyzed with the two-way ANOVA followed by Fisher’s PLSD. Significance for all tests was assumed for *p* < 0.05. Data are presented as means ± standard error (SEM).

## Results

### Glucocorticoid Receptor Translocation Into the Nucleus Induced by Acute Stress Exposure Is Blunted in TPH2^−/−^ Rats

The activation of the HPA axis after acute restraint stress results in the release of corticosterone from the adrenal glands and subsequent response in the extrinsic HPA axis structures, such as PFC *via* activation of the genomic pathway of the GRs (Adzic et al., 2009). In particular, the binding of the hormone to its receptor induces its translocation into the nucleus (Revollo and Cidlowski, 2009).

**Figure 1:**
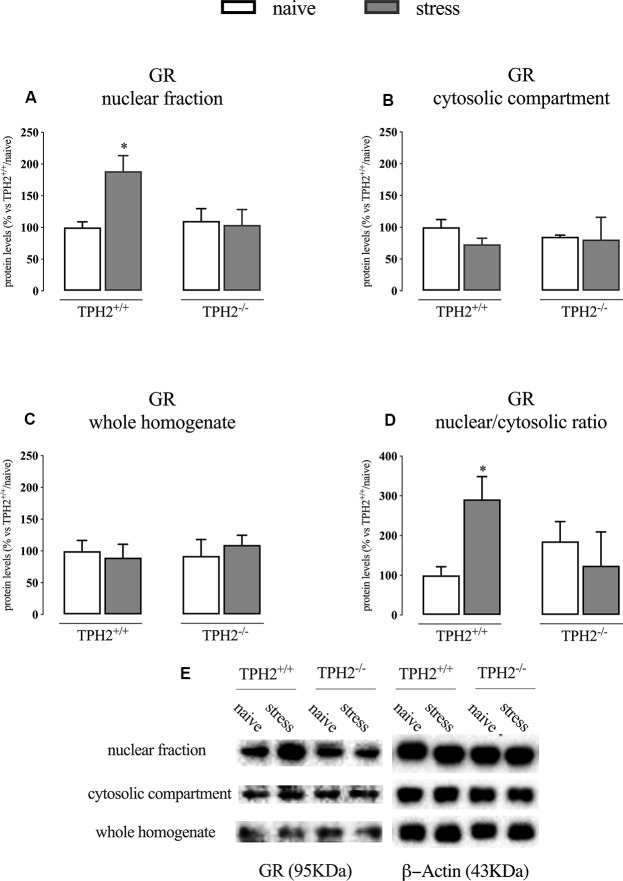
Analysis of GR protein levels in the nuclear fraction **(A)**, in the cytosolic compartment **(B)** in the whole homogenate **(C)** and the ratio between nuclear vs. cytosolic compartment **(D)** of TPH2^+/+^ and TPH2^−/−^ stressed rats. **(E)** Representative western blot bands of GR. β-actin was used as an internal control. The data are expressed as a percentage of TPH2^+/+^/naïve (set at 100%) and are represented as mean ± SEM of independent determinations. *p < 0.05 vs. TPH2^+/+^/naïve (two-way ANOVA with Fisher’s PLSD).

We first analyzed the GR protein levels in the nuclear and cytosolic fractions immediately after the end of the acute stress. As shown in Figure 1A; we found a significant genotype × stress interaction (*F*_(1,16)_ = 4.971 *p* < 0.05; two-way ANOVA) in GR protein levels in the nuclear compartment, but no effects of the genotype (*F*_(1,16)_ = 3.055 *p* > 0.05; two-way ANOVA) and of the stress (*F*_(1,16)_ = 3.747 *p* > 0.05; two-way ANOVA). Indeed, the receptor was significantly up-regulated by the stress in the nuclear compartment of TPH2^+/+^ rats (+89% *p* < 0.05 vs. TPH2^+/+^/naïve; Fisher’s PLSD), but not in the TPH2^−/−^ counterpart.

On the contrary, we did not find any significant modulation of GR protein levels in the whole homogenate and the cytosolic compartment even if a slight decrease of its levels was observed in the cytosol of TPH2^+/+^ stressed rats (−27% *p* > 0.05 vs. TPH2^+/+^/naïve; Fisher’s PLSD) (Figures 1B,C).

Finally, we looked at the ratio of the nuclear vs. cytosolic GR levels as an indicator of the translocation into the nucleus of the receptor after stress exposure and we found a borderline significance for the genotype × stress interaction (*F*_(1,13)_ = 4.761 *p* = 0.054; two-way ANOVA). Indeed, following the results described in panels A and B, we observed an increased translocation in TPH2^+/+^ stressed animals (+191% *p* < 0.05 vs. TPH2^+/+^/naïve) while this effect was blunted in TPH2^−/−^ rats (Figure 1D). Figure 1E is a representative of western blot bands.

### MAZ1 Transcription Factor Is Increased After Stress Exposure in TPH2^+/+^ but Not in TPH2^−/−^ Stressed Rats

Once in the nucleus, GRs bind the GREs, and this binding is facilitated by the presence of cofactors such as MAZ1 and SP1 (Datson et al., 2011).

**Figure 2:**
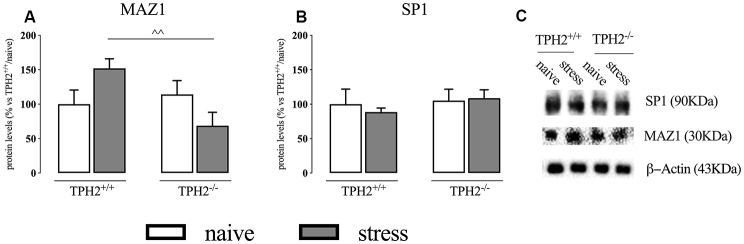
Analysis of MAZ1 **(A)** and SP1 **(B)** protein levels in the whole homogenate of TPH2^+/+^ and TPH2^−/−^ stressed rats. **(C)** Representative western blot bands of MAZ1 and SP1. β-actin was used as an internal control. The data are expressed as a percentage of TPH2^+/+^/naïve (set at 100%) and are represented as mean ± SEM of independent determinations. ^∧∧^*p* < 0.01 vs. TPH2^+/+^/stress (two-way ANOVA with Fisher’s PLSD).

In the whole homogenate, MAZ1 protein levels showed a significant genotype × stress interaction (*F*_(1,15)_ = 6.424 *p* < 0.05; two-way ANOVA) but no effect of the genotype (*F*_(1,15)_ = 3.216 *p* > 0.05; two-way ANOVA) and of the stress (*F*_(1,15)_ = 0.029 *p* > 0.05; two-way ANOVA). Accordingly, we observed a significant increase of MAZ1 protein levels in TPH2^+/+^ rats exposed to stress when compared to TPH2^−/−^ stressed animals (+121%, *p* < 0.01 vs. TPH2^−/−^/stress; Fisher’s PLSD) (Figure 2A). We did not find any changes in SP1 protein levels (Figure 2B). Figure 2C is a representative of western blot bands.

### Up-Regulation of Glucocorticoid Responsive Gene Expression Is Blunted in TPH2^−/−^ Stressed Rats

The translocation into the nucleus of the GRs leads to the expression of the glucocorticoid responsive genes, carrying the GREs.

**Figure 3:**
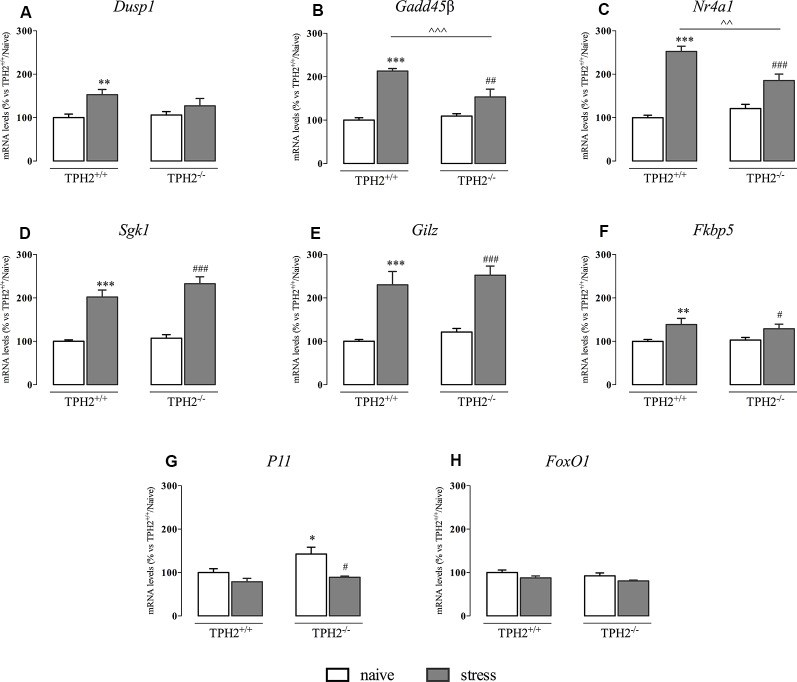
Analysis of glucocorticoid responsive gene expression in TPH2^+/+^and TPH2^−/−^ stressed rats. The data are expressed as percentage of TPH2^+/+^/naïve (set at 100%) and are represented as mean ± SEM of independent determinations. *p < 0.05; **p < 0.01; ***p < 0.001 vs. TPH2^+/+^/naïve; ^#^*p* < 0.05; ^##^*p* < 0.01; ^###^*p* < 0.001 vs. TPH2^−/−^/naïve; ^∧∧^*p* < 0.01; ^∧∧∧^*p* < 0.001 vs. TPH2^+/+^/stress (two-way ANOVA with Fisher’s PLSD).

As shown in Figure 3A, *Dusp1* mRNA levels were significantly affected by the stress (*F*_(1,33)_ = 10.770 *p* < 0.01, two-way ANOVA), that induced an increase in the expression of this gene specifically in TPH2^+/+^ rats (+53%, *p* < 0.01 vs. TPH2^+/+^/naïve).

Moreover, *Gadd45β* expression (Figure 3B) was significantly affected both by the genotype and by the stress (genotype: *F*_(1,34)_ = 8.765 *p* < 0.05 and stress: *F*_(1,34)_ = 85.447 *p* < 0.001). Indeed, stress exposure induced an increased transcription not only in TPH2^+/+^ rats (+113% *p* < 0.001 vs. TPH2^+/+^/naïve) but also in TPH2^−/−^ animals (+40%, *p* < 0.01 vs. TPH2^−/−^/naïve). However, as demonstrated by the significant genotype × stress interaction (*F*_(1,34)_ = 16.447 *p* < 0.001), the up-regulation due to the challenge found in TPH2^+/+^ was more robust (+39%, *p* < 0.001 vs. TPH2^+/+^/stress) compared to those found in TPH2^−/−^ stressed rats.

Similarly, *Nr4a1* was affected by the genotype (*F*_(1,34)_ = 4.602 *p* < 0.05, two-way ANOVA) and by the stress (*F*_(1,34)_ = 101.764 *p* < 0.001, two-way ANOVA) with a significant interaction between the two variables (*F*_(1,34)_ = 16.650 *p* < 0.001, two-way ANOVA). Accordingly, *Nr4a1* expression was up-regulated both in TPH2^+/+^ (+153% vs. TPH2^+/+^/naïve) and in TPH2^−/−^ (+54% vs. TPH2^−/−^/naïve) in comparison to their controls. Again, we observed a higher expression of *Nr4a1* in TPH2^+/+^ in comparison to TPH2^−/−^ stressed rats (+36% *p* < 0.001 vs. TPH2^+/+^/stress) (Figure 3C).

On the contrary, *Sgk1*, *Gilz* and *Fkbp5* mRNA levels were affected only by the stress (*Sgk1*: *F*_(1,35)_ = 135.512 *p* < 0.001, two-way ANOVA; *Gilz*: *F*_(1,35)_ = 96.360 *p* < 0.001, two-way ANOVA; *Fkbp5*: *F*_(1,33)_ = 16.167 *p* < 0.001, two-way ANOVA). In particular, we observed a comparable increase of these genes expression after the acute challenge both in TPH2^+/+^ (*Sgk1*: +102% *p* < 0.001; *Gilz*: +130% *p* < 0.001; *Fkbp5*: +39% *p* < 0.01 vs. TPH2^+/+^/naïve) and TPH2^−/−^ rats (*Sgk1*: +117% *p* < 0.001; *Gilz*: +107% *p* < 0.001; *Fkbp5*: +25% *p* < 0.05 vs. TPH2^−/−^/naïve) (Figures 3D–F).

Also, *P11* mRNA levels were affected by the stress (*F*_(1,35)_ = 6.158 *p* < 0.05, two-way ANOVA). However, stress exposure induced a reduction in *P11* expression in both genotypes after stress exposure (TPH2^+/+^: −21% *p* > 0.05 vs. TPH2^+/+^/naïve; TPH2^−/−^: −28% *p* < 0.05 vs. TPH2^−/−^/naïve). Moreover, TPH2^−/−^ rats showed increased levels of *P11* mRNA when compared to TPH2^+/+^(+43% *p* < 0.05 vs. TPH2^+/+^/naïve; Fisher’s PLSD) (Figure 3G).

Finally, as shown in Figure 3H, we did not find any changes in *FoxO1* expression.

### Serotonin Deficiency Alters Clock Gene Expression Profile

It has been demonstrated that acute stress exposure can influence the expression of *Per1* thanks to the presence of a canonical GRE (Yamamoto et al., 2005). Hence, seen the role of *Per1* in the control of circadian rhythms, we measured the expression of the main components of the clock machinery.

**Figure 4:**
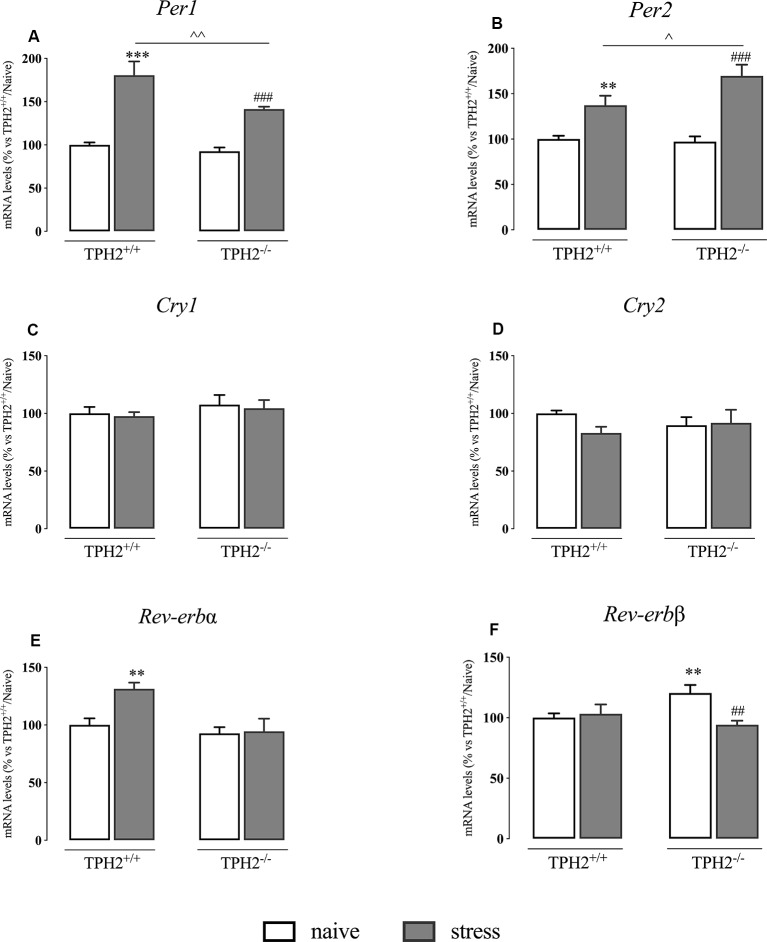
Analysis of clock genes expression in TPH2^+/+^and TPH2^−/−^ stressed rats. The data are expressed as percentage of TPH2^+/+^/naïve (set at 100%) and are represented as mean ± SEM of independent determinations. **p < 0.01; ***p < 0.001 vs. TPH2^+/+^/naïve; ^##^*p* < 0.01; ^###^*p* < 0.001 vs. TPH2^−/−^/naïve; ^∧^*p* < 0.05; ^∧∧^*p* < 0.01; vs. TPH2^+/+^/stress (two-way ANOVA with Fisher’s PLSD).

In our animals, we found a significant genotype × stress interaction (*F*_(1,35)_ = 7.098 *p* < 0.05, two-way ANOVA) and a significant effect of the genotype (*F*_(1,35)_ = 15.130 *p* < 0.01, two-way ANOVA) and of the stress (*F*_(1,35)_ = 115.534 *p* < 0.001, two-way ANOVA) on *Per1* expression. Indeed, as shown in Figure 4A, *Per1* mRNA levels were significantly up-regulated in TPH2^+/+^ stressed rats in comparison to their naïve controls (+80% *p* < 0.001 vs. TPH2^+/+^/naïve; Fisher’s PLSD). This increase was present also in TPH2^−/−^ rats exposed to the restraint stress (+52% *p* < 0.001 vs. TPH2^−/−^/naïve; Fisher’s PLSD) even if the effect was more pronounced in TPH2^+/+^ animals (+28% *p* < 0.01 vs. TPH2^+/+^/stress; Fisher’s PLSD).

Also, the expression of *Per2* showed a significant genotype × stress interaction (*F*_(1,34)_ = 5.725 *p* < 0.05, two-way ANOVA) and a significant effect of the stress (*F*_(1,34)_ = 55.671 *p* < 0.001, two-way ANOVA). However, *Per2* expression was increased more in TPH2^−/−^ stressed animals than in TPH2^+/+^ (TPH2^+/+^: +37% *p* < 0.01 vs. TPH2^+/+^/stress; TPH2^−/−^: +75% *p* < 0.001; Fisher’s PLSD). Indeed, the increased transcription of *Per2* due to stress exposure was higher in TPH2^−/−^ compared to TPH2^+/+^ animals (+24% *p* < 0.05 vs. TPH2^+/+^/stress; Fisher’s PLSD) (Figure 4B).

We did not find any changes in *Cry1* and *Cry2* expression (Figures 4C,D).

Similarly to what was observed in *Per1* expression, *Rev-erbα* mRNA levels were affected by the genotype and by the stress with a significant genotype × stress interaction (genotype: *F*_(1,35)_ = 10.544 *p* < 0.05; stress: *F*_(1,35)_ = 6.112; genotype × stress interaction: *F*_(1,35)_ = 4.984 *p* < 0.05; *p* < 0.05 two-way ANOVA). Specifically, *Rev-erbα* expression was increased in TPH2^+/+^ stressed animals (+31% *p* < 0.01 vs. TPH2^+/+^/naïve; Fisher’s PLSD) while we did not observe any changes in TPH2^−/−^ rats (Figure 4E).

Finally, as shown in Figure 4F, *Rev-erbβ* expression showed a significant genotype × stress interaction (*F*_(1,34)_ = 4.789 *p* < 0.05 two-way ANOVA). Indeed, we observed an up-regulation in *Rev-erbβ* expression in TPH2^−/−^ in comparison to TPH2^+/+^ counterpart (+20% *p* < 0.01 vs. TPH2^+/+^/naïve; Fisher’s PLSD). Moreover, *Rev-erbβ* levels were normalized in TPH2^−/−^ rats after the stress (−22% *p* < 0.01 vs. TPH2^−/−^/naïve; Fisher’s PLSD).

## Discussion

Our results show that serotonin deficiency interferes with the activation of the genomic pathway of GRs in response to an acute challenge. Moreover, it modulates the transcription of clock genes suggesting a possible alteration in circadian rhythms under stress challenge.

Interestingly, serotonin plays a crucial role in controlling brain functions not only at the basal level, but also in more dynamic situations. Indeed, we already demonstrated that the absence of serotonin in TPH2-deficient rats modulates neuroplastic mechanisms not only in basal conditions but also after acute stress exposure (Brivio et al., 2018).

The activation of the HPA axis is one of the first responses to a stressful experience. However, the downstream pathway that is activated after the release of corticosterone is very complex. In particular, once released from the adrenal glands, it targets the brain where it acts on its receptors widely localized in the cells. While membrane-bound GRs induce a rapid effect by modulating the release of neurotransmitters and mitochondrial activity through the so-called non-genomic pathway, cytosolic receptors are internalized into the nucleus where after the binding to GREs, they control the transcription of target genes (Schoneveld et al., 2004; Panettieri et al., 2019).

While in our previous study we found that the release of corticosterone following an acute challenge was independent of the serotonin levels in the brain (Brivio et al., 2018), here, we demonstrated that the internalization into the nucleus of the GRs induced by the exposure to an acute stressor happened specifically in TPH2^+/+^, but not in TPH2^−/−^ rats. This suggests that the absence of serotonin in the brain does not influence the release of the hormone from the adrenal glands, but it interferes with the activation of the genomic pathway at the central level. In line, it has been demonstrated that the modulation of the serotonergic system by chronic treatment with antidepressant drugs enhances the translocation of GRs to the nuclear compartment (Molteni et al., 2009).

By investigating the levels of GR in the different subcellular compartments, we found that the lack of serotonin inhibits stress-induced translocation of GR to the nucleus. Since the direct influence of central serotonin on corticosterone release after acute stress is excluded by our previous experiments, another mechanism linking serotonin and GR functionality should exist. Most likely the cross-talk between these two systems appears at the level of interaction between downstream pathways of serotonin receptors with GR phosphorylation. Indeed, it has been demonstrated that the entry of GR to the nucleus is facilitated by the phosphorylation of specific serines (Wang et al., 2002) *via* activation of MAPKs and CDKs pathways (Galliher-Beckley and Cidlowski, 2009). Since the activity of the MAPKs is modulated by 5-HT2A and 5HT1A receptors (Banes et al., 1999; Errico et al., 2001), blunted activation of 5-HT receptor signaling in the absence of ligand may be responsible for the lack of GR phosphorylation and its subsequent translocation to the nucleus upon acute stress. Furthermore, it has been shown that GR phosphorylation is also controlled by BDNF signaling (Arango-Lievano et al., 2015). We previously demonstrated that TPH2^−/−^ rats showed a reduced *Bdnf* expression after the exposure to 1 h of acute restraint stress compared to the wild type counterpart (Brivio et al., 2018). This may lead to a decreased ability of the neurotrophin-related pathways to phosphorylate GRs.

Interestingly, the GREs are characterized by the presence of GC enriched binding sites that are targeted by transcription factors such as MAZ1 and SP1. These transcription factors in the proximity to GREs can potentiate the GR binding and therefore, enhance the transcriptional activation of target genes (Datson et al., 2011). Here, in line with the increase of GR in the nucleus, we observed increased levels of MAZ1 in TPH2^+/+^ stressed animals contrasting with a slight decrease in TPH2^−/−^ stressed animals. This data suggests that the reduced activation of the genomic pathway in TPH2^−/−^ rats in response to the acute challenge may be caused not only by the lack of GRs translocation *per se* but also by the reduced levels of this cofactor. On the contrary, SP1 was not altered after stress exposure. Hence, these two cofactors may influence the interaction between GRs and their responsive elements with different timing. Furthermore, even if both MAZ1 and SP1 can interact with GRs, SP1 seems to be mainly related to mineralocorticoid receptors (Meinel et al., 2013) suggesting its potential implication in physiological conditions and not in response to an acute challenge.

The GR, as a nuclear receptor, acts as a transcriptional regulator interfering with the transcription of genes carrying the GRE in their regulatory sequence (Gray et al., 2017). In line, we recently demonstrated that the translocation into the nucleus is paralleled by an up-regulation of the expression of genes containing the GRE such as *Gadd45β*, *Sgk1*, *Nr4a1*, and *Dusp1* (Calabrese et al., 2020). Here, we observed that GR responsive genes were activated more in TPH2^+/+^ than in TPH2^−/−^ rats. In particular, *Dusp1* mRNA was increased specifically in TPH2^+/+^ while no changes were observed in TPH2^−/−^ rats. Similarly, *Gadd45β* and *Sgk1* mRNAs were increased in both genotypes, but more in TPH2^+/+^ than in TPH2^−/−^ rats. This suggests the ability of serotonin to interfere with their transcription. Indeed, several studies showed that pharmacological or genetic alterations of the serotonergic system can modulate their expression (Gonzalez-Nicolini and McGinty, 2002; Duric et al., 2010; Calabrese et al., 2013). However, other glucocorticoid responsive genes were equally upregulated after the acute challenge in the two genotypes. This could be due to other factors that can influence their expression and that may contribute to the stress response (Lang et al., 2014; Zannas et al., 2016). Furthermore, it could not be excluded that the absence of serotonin may modify the epigenetic make-up of some GR related genes *via* the histone-serotonylation mechanism (Farrelly et al., 2019), and therefore their transcription after an acute challenge.

As mentioned, serotonin is involved in the control of various physiological functions and, among them, the circadian system is braided with the neurotransmitter both anatomically and genetically (Deurveilher and Semba, 2005). Furthermore, it has been demonstrated that genes involved in the clock gene machinery can be affected by acute stress exposure (Yamamoto et al., 2005). Interestingly, *Per1* expression showed a similar pattern as *Gadd45β* and *Nr4a1*, with increased transcription in TPH2^+/+^ compared to TPH2^−/−^ rats exposed to stress. On the contrary, *Per2* mRNA levels were increased more in TPH2^−/−^ than in TPH2^+/+^ rats. This different pattern could be due to a different responsiveness of *Per1* and *Per2* to GRs. Indeed, while *Per1* carries a canonical GRE, *Per2* has an intronic binding sequence that confers GR responsiveness to the gene (So et al., 2009). Moreover, *Rev-erbα* expression was up-regulated by stress in TPH2^+/+^ but not in TPH2^−/−^ rats while *Rev-erbβ* expression showed a basal increase in TPH2^−/−^ rats while a normalization occurred after the acute stress. Since *Rev-erbs* are responsible for the proper interlocking feedback loop in the clock machinery (Lowrey and Takahashi, 2011), the modulations observed could indicate alterations in the circadian rhythm at basal level and in the feedback to clock gene activation after the stress.

All in all, our data confirm the involvement of serotonin in the proper stress response and that the absence of the neurotransmitter in the brain interferes with the functionality of the HPA axis in the PFC modulating the genomic pathway activation. Similarly, serotonin depletion affected clock gene activity at basal level and in response to the acute challenge, suggesting a potential link between serotonin, stress and circadian rhythms.

## Data Availability Statement

The datasets generated for this study are available on request to the corresponding author.

## Ethics Statement

The animal study was reviewed and approved by LAGeSo, Berlin, Germany.

## Author Contributions

FC and NA: conception and study design. GS, PB, PP, and MT: performed experiments. GS, PB, FC, and NA: performed data analysis and interpretation of the data. GS: drafted the manuscript. FC, NA, and MB critically revised the manuscript. All authors critically reviewed the content and approved the final version for publication.

## Conflict of Interest

The authors declare that the research was conducted in the absence of any commercial or financial relationships that could be construed as a potential conflict of interest.

## References

[B1] Adzic M. Djordjevic J. Djordjevic A. Niciforovic A. Demonacos C. Radojcic M.. (2009). Acute or chronic stress induce cell compartment-specific phosphorylation of glucocorticoid receptor and alter its transcriptional activity in Wistar rat brain. J. Endocrinol. 202, 87–97. 10.1677/joe-08-0509 19406955 PMC2695659

[B2] Akiskal H. S. McKinney W. T. (1973). Psychiatry and pseudopsychiatry. Arch. Gen. Psychiatry 28, 367–373. 10.1001/archpsyc.1973.01750330055010 4688626

[B3] Akiskal H. S. McKinney W. T. Jr. (1975). Overview of recent research in depression: integration of ten conceptual models into a comprehensive clinical frame. Arch. Gen. Psychiatry 32, 285–305. 10.1001/archpsyc.1975.01760210019001 1092281

[B4] Arango-Lievano M. Lambert W. M. Bath K. G. Garabedian M. J. Chao M. V. Jeanneteau F. (2015). Neurotrophic-priming of glucocorticoid receptor signaling is essential for neuronal plasticity to stress and antidepressant treatment. Proc. Natl. Acad. Sci. U S A 112, 15737–15742. 10.1073/pnas.1509045112 26630005 PMC4697403

[B5] Banes A. Florian J. A. Watts S. W. (1999). Mechanisms of 5-hydroxytryptamine(2A) receptor activation of the mitogen-activated protein kinase pathway in vascular smooth muscle. J. Pharmacol. Exp. Ther. 291, 1179–1187. 10565840

[B6] Breslau N. Davis G. C. (1986). Chronic stress and major depression. Arch. Gen. Psychiatry 43, 309–314. 10.1001/archpsyc.1986.01800040015003 2937384

[B7] Brivio P. Corsini G. Riva M. A. Calabrese F. (2019). Chronic vortioxetine treatment improves the responsiveness to an acute stress acting through the ventral hippocampus in a glucocorticoid-dependent way. Pharmacol. Res. 142, 14–21. 10.1016/j.phrs.2019.02.006 30735803

[B8] Brivio P. Sbrini G. Peeva P. Todiras M. Bader M. Alenina N.. (2018). TPH2 deficiency influences neuroplastic mechanisms and alters the response to an acute stress in a sex specific manner. Front. Mol. Neurosci. 11:389. 10.3389/fnmol.2018.00389 30425618 PMC6218558

[B9] Brivio P. Sbrini G. Riva M. A. Calabrese F. (2020). Acute stress induces cognitive improvement in the novel object recognition task by transiently modulating bdnf in the prefrontal cortex of male rats. Cell. Mol. Neurobiol. [Epub ahead of print]. 10.1007/s10571-020-00793-7 31960229 PMC11448843

[B10] Calabrese F. Brivio P. Sbrini G. Gruca P. Lason M. Litwa E.. (2020). Effect of lurasidone treatment on chronic mild stress-induced behavioural deficits in male rats: the potential role for glucocorticoid receptor signalling. J. Psychopharmacol. 34, 420–428. 10.1177/0269881119895547 31913065

[B11] Calabrese F. Guidotti G. Middelman A. Racagni G. Homberg J. Riva M. A. (2013). Lack of serotonin transporter alters BDNF expression in the rat brain during early postnatal development. Mol. Neurobiol. 48, 244–256. 10.1007/s12035-013-8449-z 23564488

[B12] Caspi A. Sugden K. Moffitt T. E. Taylor A. Craig I. W. Harrington H. L.. (2003). Influence of life stress on depression: moderation by a polymorphism in the 5-HTT gene. Science 301, 386–389. 10.1126/science.1083968 12869766

[B13] Datson N. A. Polman J. A. E. de Jonge R. T. van Boheemen P. T. M. van Maanen E. M. T. Welten J.. (2011). Specific regulatory motifs predict glucocorticoid responsiveness of hippocampal gene expression. Endocrinology 152, 3749–3757. 10.1210/en.2011-0287 21846803

[B14] Deurveilher S. Semba K. (2005). Indirect projections from the suprachiasmatic nucleus to major arousal-promoting cell groups in rat: implications for the circadian control of behavioural state. Neuroscience 130, 165–183. 10.1016/j.neuroscience.2004.08.030 15561433

[B15] Duman R. S. Monteggia L. M. (2006). A neurotrophic model for stress-related mood disorders. Biol. Psychiatry 59, 1116–1127. 10.1016/j.biopsych.2006.02.013 16631126

[B16] Duric V. Banasr M. Licznerski P. Schmidt H. D. Stockmeier C. A. Simen A. A.. (2010). A negative regulator of MAP kinase causes depressive behavior. Nat. Med. 16, 1328–1332. 10.1038/nm.2219 20953200 PMC3066515

[B17] Errico M. Crozier R. A. Plummer M. R. Cowen D. S. (2001). 5-HT_7_ receptors activate the mitogen activated protein kinase extracellular signal related kinase in cultured rat hippocampal neurons. Neuroscience 102, 361–367. 10.1016/s0306-4522(00)00460-7 11166122

[B18] Farrelly L. A. Thompson R. E. Zhao S. Lepack A. E. Lyu Y. Bhanu N. V.. (2019). Histone serotonylation is a permissive modification that enhances TFIID binding to H3K4me3. Nature 567, 535–539. 10.1038/s41586-019-1024-7 30867594 PMC6557285

[B19] Fitzpatrick P. F. (1999). Tetrahydropterin-dependent amino acid hydroxylases. Annu. Rev. Biochem. 68, 355–381. 10.1146/annurev.biochem.68.1.355 10872454

[B20] Galliher-Beckley A. J. Cidlowski J. A. (2009). Emerging roles of glucocorticoid receptor phosphorylation in modulating glucocorticoid hormone action in health and disease. IUBMB Life 61, 979–986. 10.1002/iub.245 19787703

[B21] Gonzalez-Nicolini V. McGinty J. F. (2002). Gene expression profile from the striatum of amphetamine-treated rats: a cDNA array and *in situ* hybridization histochemical study. Brain Res. Gene Expr. Patterns 1, 193–198. 10.1016/s1567-133x(02)00017-0 12638131

[B22] Gray J. D. Kogan J. F. Marrocco J. McEwen B. S. (2017). Genomic and epigenomic mechanisms of glucocorticoids in the brain. Nat. Rev. Endocrinol. 13, 661–673. 10.1038/nrendo.2017.97 28862266

[B23] Herman J. P. Figueiredo H. Mueller N. K. Ulrich-Lai Y. Ostrander M. M. Choi D. C.. (2003). Central mechanisms of stress integration: hierarchical circuitry controlling hypothalamo-pituitary-adrenocortical responsiveness. Front. Neuroendocrinol. 24, 151–180. 10.1016/j.yfrne.2003.07.001 14596810

[B24] Kaplan K. Echert A. E. Massat B. Puissant M. M. Palygin O. Geurts A. M.. (2016). Chronic central serotonin depletion attenuates ventilation and body temperature in young but not adult Tph2 knockout rats. J. Appl. Physiol. 120, 1070–1081. 10.1152/japplphysiol.01015.2015 26869713 PMC4855209

[B25] Lang F. Stournaras C. Alesutan I. (2014). Regulation of transport across cell membranes by the serum-and glucocorticoid-inducible kinase SGK1. Mol. Membr. Biol. 31, 29–36. 10.3109/09687688.2013.874598 24417516

[B26] Lowrey P. L. Takahashi J. S. (2011). Genetics of circadian rhythms in mammalian model organisms. Adv. Genet. 74, 175–230. 10.1016/b978-0-12-387690-4.00006-4 21924978 PMC3709251

[B27] Lupien S. J. Juster R. P. Raymond C. Marin M. F. (2018). The effects of chronic stress on the human brain: from neurotoxicity, to vulnerability, to opportunity. Front. Neuroendocrinol. 49, 91–105. 10.1016/j.yfrne.2018.02.001 29421159

[B28] McEwen B. S. (2007). Physiology and neurobiology of stress and adaptation: central role of the brain. Physiol. Rev. 87, 873–904. 10.1152/physrev.00041.2006 17615391

[B29] Meinel S. Ruhs S. Schumann K. Strätz N. Trenkmann K. Schreier B.. (2013). Mineralocorticoid receptor interaction with SP1 generates a new response element for pathophysiologically relevant gene expression. Nucleic Acids Res. 41, 8045–8060. 10.1093/nar/gkt581 23821666 PMC3783164

[B30] Molteni R. Calabrese F. Cattaneo A. Mancini M. Gennarelli M. Racagni G.. (2009). Acute stress responsiveness of the neurotrophin bdnf in the rat hippocampus is modulated by chronic treatment with the antidepressant duloxetine. Neuropsychopharmacology 34, 1523–1532. 10.1038/npp.2008.208 19020498

[B31] Mosienko V. Beis D. Pasqualetti M. Waider J. Matthes S. Qadri F.. (2015). Life without brain serotonin: reevaluation of serotonin function with mice deficient in brain serotonin synthesis. Behav. Brain Res. 277, 78–88. 10.1016/j.bbr.2014.06.005 24928769

[B32] Nader N. Chrousos G. P. Kino T. (2010). Interactions of the circadian CLOCK system and the HPA axis. Trends Endocrinol. Metab. 21, 277–286. 10.1016/j.tem.2009.12.011 20106676 PMC2862789

[B33] Ottenhof K. W. Sild M. Lévesque M. L. Ruhé H. G. Booij L. (2018). TPH2 polymorphisms across the spectrum of psychiatric morbidity: a systematic review and meta-analysis. Neurosci. Biobehav. Rev. 92, 29–42. 10.1016/j.neubiorev.2018.05.018 29775696

[B34] Pan L. Gilbert F. (1992). Activation of 5-HT_1A_ receptor subtype in the paraventricular nuclei of the hypothalamus induces CRH and ACTH release in the rat. Neuroendocrinology 56, 797–802. 10.1159/000126332 1369587

[B35] Panettieri R. A. Schaafsma D. Amrani Y. Koziol-White C. Ostrom R. Tliba O. (2019). Non-genomic effects of glucocorticoids: an updated view. Trends Pharmacol. Sci. 40, 38–49. 10.1016/j.tips.2018.11.002 30497693 PMC7106476

[B36] Paxinos G. Watson C. (2007). The Rat Brain in Stereotaxic Coordinates Sixth Edition. San Diego: Elsevier Academic Press.

[B37] Revollo J. R. Cidlowski J. A. (2009). Mechanisms generating diversity in glucocorticoid receptor signaling. Ann. N Y Acad. Sci. 1179, 167–178. 10.1111/j.1749-6632.2009.04986.x 19906239

[B38] Schoneveld O. J. L. M. Gaemers I. C. Lamers W. H. (2004). Mechanisms of glucocorticoid signalling. Biochim. Biophys. Acta 1680, 114–128. 10.1016/j.bbaexp.2004.09.004 15488991

[B39] So A. Y. L. Bernal T. U. Pillsbury M. L. Yamamoto K. R. Feldman B. J. (2009). Glucocorticoid regulation of the circadian clock modulates glucose homeostasis. Proc. Natl. Acad. Sci. U S A 106, 17582–17587. 10.1073/pnas.0909733106 19805059 PMC2757402

[B40] Stuss D. T. Knight R. T. (2009). Principles of Frontal Lobe Function. New York, NY: Oxford University Press.

[B41] Trapp T. Rupprecht R. Castrén M. Reul J. M. H. M. Holsboer F. (1994). Heterodimerization between mineralocorticoid and glucocorticoid receptor: a new principle of glucocorticoid action in the CNS. Neuron 13, 1457–1462. 10.1016/0896-6273(94)90431-6 7993637

[B42] Van de Kar L. D. Javed A. Zhang Y. Serres F. Raap D. K. Gray T. S. (2001). 5-HT2A receptors stimulate ACTH, corticosterone, oxytocin, renin, and prolactin release and activate hypothalamic CRF and oxytocin-expressing cells. J. Neurosci. 21, 3572–3579. 10.1523/JNEUROSCI.21-10-03572.2001 11331386 PMC6762485

[B43] Walther D. J. Peter J. U. Bashammakh S. Hörtnagl H. Voits M. Fink H.. (2003). Synthesis of serotonin by a second tryptophan hydroxylase isoform. Science 299:76. 10.1126/science.1078197 12511643

[B44] Wang Z. Frederick J. Garabedian M. J. (2002). Deciphering the phosphorylation “code” of the glucocorticoid receptor *in vivo*. J. Biol. Chem. 277, 26573–26580. 10.1074/jbc.M110530200 12000743

[B45] Yamamoto T. Nakahata Y. Tanaka M. Yoshida M. Soma H. Shinohara K.. (2005). Acute physical stress elevates mouse Period1 mRNA expression in mouse peripheral tissues *via* a glucocorticoid-responsive element. J. Biol. Chem. 280, 42036–42043. 10.1074/jbc.m509600200 16249183

[B46] Zannas A. S. Wiechmann T. Gassen N. C. Binder E. B. (2016). Gene-stress-epigenetic regulation of FKBP5: clinical and translational implications. Neuropsychopharmacology 41, 261–274. 10.1038/npp.2015.235 26250598 PMC4677131

